# Effects of disusing closed suction drainage in simultaneous bilateral total hip arthroplasty: A retrospective cohort study

**DOI:** 10.1371/journal.pone.0247845

**Published:** 2021-03-03

**Authors:** Chan-Woo Park, Seung-Jae Lim, Insun Yoo, Youngsik Lee, Jae-Yeon Won, Youn-Soo Park

**Affiliations:** Department of Orthopedic Surgery, Samsung Medical Center, Sungkyunkwan University School of Medicine, Seoul, Korea; Ohio State University Wexner Medical Center Department of Surgery, UNITED STATES

## Abstract

**Purpose:**

Increased blood loss remains a major drawback of simultaneous bilateral total hip arthroplasty (SBTHA). We examined the effects of disusing closed suction drainage (CSD) on postoperative blood loss and transfusion requirement in cementless SBTHA.

**Methods:**

A retrospective cohort study was conducted with a consecutive series of cementless SBTHAs performed by a single surgeon between January 2014 and March 2017. The surgeon routinely used CSD until May 2015 and refrained from CSD in all primary THAs thereafter. This study included SBTHAs with intravenous administration of tranexamic acid (TXA). Postoperative hemoglobin drop, blood loss, transfusion rate, pain scores, complication rates, and implant survivorships were compared between the groups of SBTHA with and without CSD. The minimum follow-up duration was 1 year.

**Results:**

Among the 110 patients (220 hips), 46 (92 hips) and 64 (128 hips) underwent SBTHA with and without CSD, respectively. Maximum hemoglobin drop (mean, 4.8 vs. 3.9 g/dL; P = 0.001), calculated blood loss (mean, 1530 vs. 1190 mL; P<0.001), transfusion rate (45.7% vs. 21.9%; P = 0.008), and length of hospital stay (mean, 6.6 vs. 5.8 days; P = 0.004) were significantly lower in patients without CSD. There were no significant differences in postoperative pain scales and wound complication rates. The mean Harris Hip scores at final follow-up (92.5 vs. 92.1; P = 0.775) and implant survivorships with an end-point of any revision at 4 years (98.9% vs. 98.4%; log-rank, P = 0.766) were similar between groups.

**Conclusions:**

Disusing CSD significantly reduced postoperative blood loss and transfusion requirement without increasing postoperative pain and surgical wound complications in cementless SBTHA with concurrent administration of intravenous TXA.

## Introduction

Many individuals requiring total hip arthroplasty (THA) have a predisposing condition, commonly affecting both hips (e.g., developmental dysplasia, osteonecrosis, rheumatoid arthritis, and ankylosing spondylitis). It has been reported that 16–85% of those undergoing unilateral THA with bilateral disease eventually required contralateral THA [1–4]. For these patients, simultaneous bilateral total hip arthroplasty (SBTHA) can be an attractive option; it offers several potential advantages over staged bilateral THA, including a single anesthesia, shorter total operation time and hospital stay, lower overall cost, reduced recovery and rehabilitation time, and earlier return to daily activities [5–9]. Recent studies have reported that SBTHA can be a safe procedure, showing similar or lower incidence of postoperative complications compared to staged operations [6, 7, 10–13]. On the other hand, there are evident drawbacks of SBTHA including more frequent requirement for allogenic transfusion [14–17].

Closed suction drainage (CSD) has been commonly used in THA to suppress wound inflammation, improve postoperative recovery, and reduce local complications such as hematoma, edema, and infection [18, 19]. Nevertheless, some surgeons disagree with the routine use of CSD in THA, as it may interfere with the natural tamponade effect and lead to increased bleeding without providing obvious benefits [20–22]. Recently, the wide spread use of intravenous or topical tranexamic acid (TXA) has significantly reduced perioperative blood loss in total joint arthroplasty (TJA) [23–27]. In combination with TXA, a meticulous hemostasis and securely layered wound closure in THA may negate the necessity for CSD. However, there are still controversies over the CSD use in THA, which largely depends on the surgeon’s preference.

To date, no study has investigated the effects of disusing CSD on postoperative blood loss and transfusion requirement in cementless SBTHA with concurrent administration of TXA. The purpose of this study was to compare postoperative hemoglobin drop, calculated blood loss, transfusion rate, patient-reported outcomes, and major complication rates between the groups of SBTHA with CSD and without CSD (non-CSD).

## Materials and methods

This study was conducted under the approval of the institutional review board (IRB) of Samsung Medical Center (IRB Number: 2020-04-128). All medical records at Samsung Medical Center were accessed from April 2020 to October 2020. As this study was a non-interventional retrospective study and all data were fully anonymized prior to access, the IRB waived the need for individual informed consent.

### Study design

This retrospective cohort study included a consecutive series of cementless SBTHAs performed by a single experienced surgeon between January 2014 and March 2017. During this period, SBTHA was considered in patients under the age of 80 years without severe medical comorbidity. Since 2014, intravenous TXA was used in all patients undergoing elective THA in our institution, unless the patient had a history of cardiovascular disease, cerebrovascular accident, thrombophlebitis, or venous thromboembolic event. The surgeon used CSD in all THAs until May 2015, and then adapted his protocol to avoid using CSD in all primary cases. Patients were excluded from the study if they used autologous transfusion system, did not receive intravenous TXA during surgery, or were lost to follow-up within the first year post surgery. All data were retrospectively analyzed using the electronic medical records. Amount of blood loss, transfusion rate, early postoperative outcomes, and final surgical outcomes of SBTHAs were compared between the CSD and the non-CSD groups.

### Surgical procedures

Spinal anesthesia was considered primarily if the patient had no bleeding tendency (e.g., known bleeding disorders, current anti-platelet or anticoagulant users) and agreed with the regional anesthesia. All operations were performed using the modified Watson-Jones anterolateral approach in a lateral position. Cementless acetabular cups and tapered wedge stems were used in all cases. After implantation, meticulous hemostasis and securely layered sutures were performed, followed by a compressive, sterile, closed wound dressing. TXA was intravenously administrated in all cases. The total dosage of TXA was adjusted for patient’s body weight (20 mg/kg). The routine TXA protocol consisted of two doses; a first dose (10 mg/kg) was administered prior to skin incision of the first hip, followed by a second dose (10 mg/kg) before wound closure of the second hip. All surgical procedures were identical in both groups except for the CSD use. In the CSD group, a 3.2 mm drain tube with a 400 mL container was placed at the end of each operation with application a full-negative pressure (90 mmHg). All CSDs were removed within 48 hours at the time of initial dressing change scheduled on the second postoperative day. The amount of drainage was not taken into account in removing CSD.

### Postoperative managements

Hemoglobin levels were measured preoperatively, immediately after surgery, and on first, third, and fifth postoperative days. Additional hemoglobin concentrations were checked as clinically indicated. The definite criteria for packed red blood cell (pRBC) transfusion was a hemoglobin level of < 8.0 g/dL. For hemoglobin levels between 8.0 and 10.0 g/dL, transfusion was considered if the patient had symptoms of anemia (dizziness, altered mental status, or shortness of breath), unexplained tachycardia or hypotension unresponsive to fluid replacement. The typical volumes of pRBC ranged from 200 to 250 ml per unit. Prophylactic antibiotics were administered intravenously for 48 hours after surgery. Thromboembolic prophylaxis included early ambulation, use of intermittent pneumatic compression device, and prescription of enteric-coated aspirin (100 mg daily) for 6 weeks. Patients were encouraged to perform ankle dorsiflexion and quadriceps strengthening exercises, commencing immediately after surgery, and ambulation was allowed with full weight-bearing from 6 hours after surgery. The routine discharge was planned between 4 and 7 postoperative days, depending on the patients’ recovery, general condition, and preference. Discharge plan was delayed if the postoperative recovery was late or complications requiring additional medical treatment occurred.

### Outcome measurements

The primary outcome measures were postoperative hemoglobin drop, calculated blood loss, amount of transfusion, in-hospital pain scores, local wound complications, and duration of hospital stay. The individual blood volume was estimated by the method previously described by Nadler et al. [28].

$$\text{Blood}\;\text{volume}\;\left(\text{BV}\right)=\text{k}1\times \text{height}\;{\left(\text{m}\right)}^{\text{3}}+\text{k}2\times \text{weight}\;\left(\text{kg}\right)+\text{k}3$$

(For men, k1 = 0.3669, k2 = 0.03219, k3 = 0.6041; for women, k1 = 0.3561, k2 = 0.03308, k3 = 0.1833)

The calculated blood loss was determined using the difference between the preoperative and the lowest postoperative hemoglobin levels, with adjustment of hemoglobin change by postoperative transfusion. Visually estimated blood loss in the operating room and postoperative drain outputs were excluded in the calculation [23].

Hbloss=(BV×Hbi−Hbl)×0.001+Hbt

Bloodloss=1000×Hbloss/Hbi

(Hbi, preoperative hemoglobin; Hbl, lowest postoperative hemoglobin; Hbt, hemoglobin change by transfusion)

The transfusion volume was calculated as the total units of intraoperative and postoperative pRBC transfusion. Postoperative pain scores were recorded at least 3 times a day using the Numerical Rating Scale (NRS, 0 [no pain] to 10 [the worst pain imaginable]) during the hospital admission. The highest NRS recorded during each day, including both hips, was used as the daily highest pain score. Local wound complications included occurrence of hematoma, prolonged oozing, superficial infection, wound dehiscence, and drain hole leakage. We defined severe hematoma as a disseminated hematoma that caused any signs of compartment syndrome or required evacuation surgery due to its progressive nature despite conservative managements [29]. All in-hospital complications were identified by reviewing the electronic progress notes and discharge abstracts recorded by orthopedic residents.

Other secondary outcome measures were major medical and surgical complications, clinical outcomes at final follow-up, and revision-free implant survivorships. Major medical complications included the occurrence of cardiovascular disease, cerebrovascular accident, respiratory disorder, and any systemic conditions requiring a transfer to the intensive care unit (ICU) or readmission within 90 days. The occurrence of venous thromboembolic event, dislocation, periprosthetic joint infection, periprosthetic femoral fracture, and aseptic loosening of implants were considered major surgical complications. The combined range of motion (ROM) was determined as the sum of degrees in flexion to extension, adduction to abduction, and internal-to-external rotation of the hip [30]. Clinical scores were assessed using the Harris Hip Score (HHS) system and University of California, Los Angeles (UCLA) activity score.

### Statistical analysis

Continuous variables in the two groups were compared using Student’s t-tests or Wilcoxon rank-sum tests. Differences in the distribution of categorical variables were determined using Chi-squared or Fisher’s exact tests. Kaplan-Meier survival analysis was used to estimate implant survivorships and an intergroup difference was determined by the log-rank test. All data analyses were performed using SPSS Statistics version 25.0 software (IBM Corp., Armonk, NY). In all analyses, a P-value <0.05 was considered statistically significant.

## Results

### Baseline information

Among the 121 patients (242 hips) who underwent SBTHA during the inclusion period, 110 patients (220 hips) were finally included in this study. Of them, 46 patients (92 hips) underwent SBTHA with CSD from January 2014 to May 2015, while 64 patients (128 hips) underwent SBTHA without CSD from June 2015 to March 2017. Among the 110 patients, 52 (47.3%) were female. The mean age and body mass index (BMI) at the time of surgery were 48.7 ± 13.6 years and 24.4 ± 4.0 kg/m^2^, respectively. The most common preoperative diagnosis was osteonecrosis of the femoral head (65.5%). There were no significant differences in the baseline information and surgical characteristics between the CSD and the non-CSD groups (Table 1). The mean duration of follow-up was 4 (range, 1–6) years.

**Table 1:** Baseline information and surgical characteristics.

Characteristic	CSD	non-CSD	P-value
Number of patients (hips)	46 (92)	64 (128)	
Age* (yr)	48.4 ± 13.3	48.9 ± 14.0	0.860
Female sex	21 (45.7%)	31 (48.4%)	0.773
Body mass index* (kg/m^2^)	24.5 ± 4.1	24.3 ± 3.9	0.778
ASA physical status classification			0.825
I	23 (50.0%)	28 (43.8%)	
II	20 (43.5%)	32 (50.0%)	
III	3 (6.5%)	4 (6.3%)	
Diagnosis			0.873
Osteonecrosis of the femoral head	29 (63.0%)	43 (67.2%)	
Secondary osteoarthritis	9 (19.6%)	12 (18.8%)	
Primary osteoarthritis	8 (17.4%)	9 (14.1%)	
Harris Hip Score*	40.3 ± 11.7	39.8 ± 13.7	0.831
UCLA activity score†	3 (1–5)	3 (1–5)	0.550
Combined range of motion* (°)	157.2 ± 47.3	150.7 ± 39.3	0.274
Type of anesthesia			0.805
Spinal	38 (82.6%)	54 (84.4%)	
General	8 (17.4%)	10 (15.6%)	
Operation time (min)	143.4 ± 20.9	139.7 ± 21.5	0.382

*The values are given as the mean and the standard deviation.

†The values are given as the median with the range in parentheses. Other values are given as the number of patients with the percentage in parentheses. CSD, closed suction drainage; ASA, American Society of Anesthesiologists; UCLA, University of California, Los Angeles.

### Blood loss and transfusion

Preoperative hemoglobin level and estimated blood volume were not significantly different between the patients of the 2 groups. Maximum postoperative hemoglobin drop (mean, 4.8 vs. 3.9 g/dL; P = 0.001) and calculated blood loss (mean, 1530.1 vs. 1190.4 mL; P<0.001) were significantly lower in the non-CSD group (Table 2). The rate (45.7% vs. 21.9%; P = 0.008) and the mean units of pRBC transfusion (0.9 vs. 0.4; P = 0.005) were also lower in the non-CSD group. For the CSD group, the total drain output from both hips was measured 1084.9 ± 480.5 mL.

**Table 2:** Hemoglobin level, blood loss, and transfusion.

Parameter	CSD	non-CSD	P-value
Estimated blood volume (mL)	4161.5 ± 103.8	4085.8 ± 99.5	0.607
Hemoglobin			
Preoperative level (g/dL)	13.6 ± 1.5	13.6 ± 1.4	0.838
Lowest postoperative level (g/dL)	8.8 ± 1.1	9.8 ± 1.2	<0.001
Maximum drop (g/dL)	4.8 ± 1.6	3.9 ± 1.1	0.001
Calculated blood loss (mL)	1530.1 ± 441.6	1190.4 ± 362.1	<0.001
Packed red blood cell			
Number of patients with transfusion*	21 (45.7%)	14 (21.9%)	0.008
Amount of transfusion (Unit)	0.9 ± 1.2	0.4 ± 0.7	0.005
Total drain output (mL)	1084.9 ± 480.5		

*The values are given as the number of patients with the percentage in parentheses. Other values are given as the mean and the standard deviation. CSD, closed suction drainage.

### Early postoperative results

Postoperative daily pain scales were not significantly different between the CSD and non-CSD groups throughout the hospital admission (Table 3). No skin necrosis, severe hematoma, nerve palsy, or wound dehiscence was observed in either group. Prolonged wound oozing was identified with similar rates (4.3% vs. 3.9%) in the 2 groups, whereas superficial wound infection occurred only in the CSD group (2 hips, 2.2%). Prolonged drain hole leakage was found in 5 hips (5.4%) in the CSD group. There was no significant difference in the wound complication rates between the first and second hips of SBTHA in either group. The mean length of hospital stay was shorter in the non-CSD group (6.6 vs. 5.8 days; P = 0.004).

**Table 3:** Early postoperative pain scales and complications.

Parameter	CSD	non-CSD	P-value
Daily highest pain score* (NRS)			
POD 1	4.5 ± 1.7	4.4 ± 1.6	0.737
POD 2	3.7 ± 1.3	3.5 ± 1.7	0.378
POD 3	3.4 ± 1.5	3.3 ± 1.7	0.706
POD 4	3.0 ± 1.4	2.8 ± 1.4	0.552
Nerve palsy	0 (0%)	0 (0%)	1.000
Local wound complication			
Skin necrosis	0 (0%)	0 (0%)	1.000
Wound dehiscence	0 (0%)	0 (0%)	1.000
Severe hematoma	0 (0%)	0 (0%)	1.000
Prolonged oozing	4 (4.3%)	5 (3.9%)	1.000
Superficial infection	2 (2.2%)	0 (0%)	0.174
Prolonged drain hole leakage	5 (5.4%)		
Major medical complication†	0 (0%)	0 (0%)	1.000
Length of hospital stay* (days)	6.6 ± 1.1	5.8 ± 1.4	0.004

*The values are given as the mean and the standard deviation.

†The values are given as the number of patients with the percentage in parentheses. Other values are given as the number of hips with the percentage in parentheses. CSD, closed suction drainage; NRS, Numerical Rating Scale; POD, postoperative day.

### Final surgical outcomes

**Fig 1:**
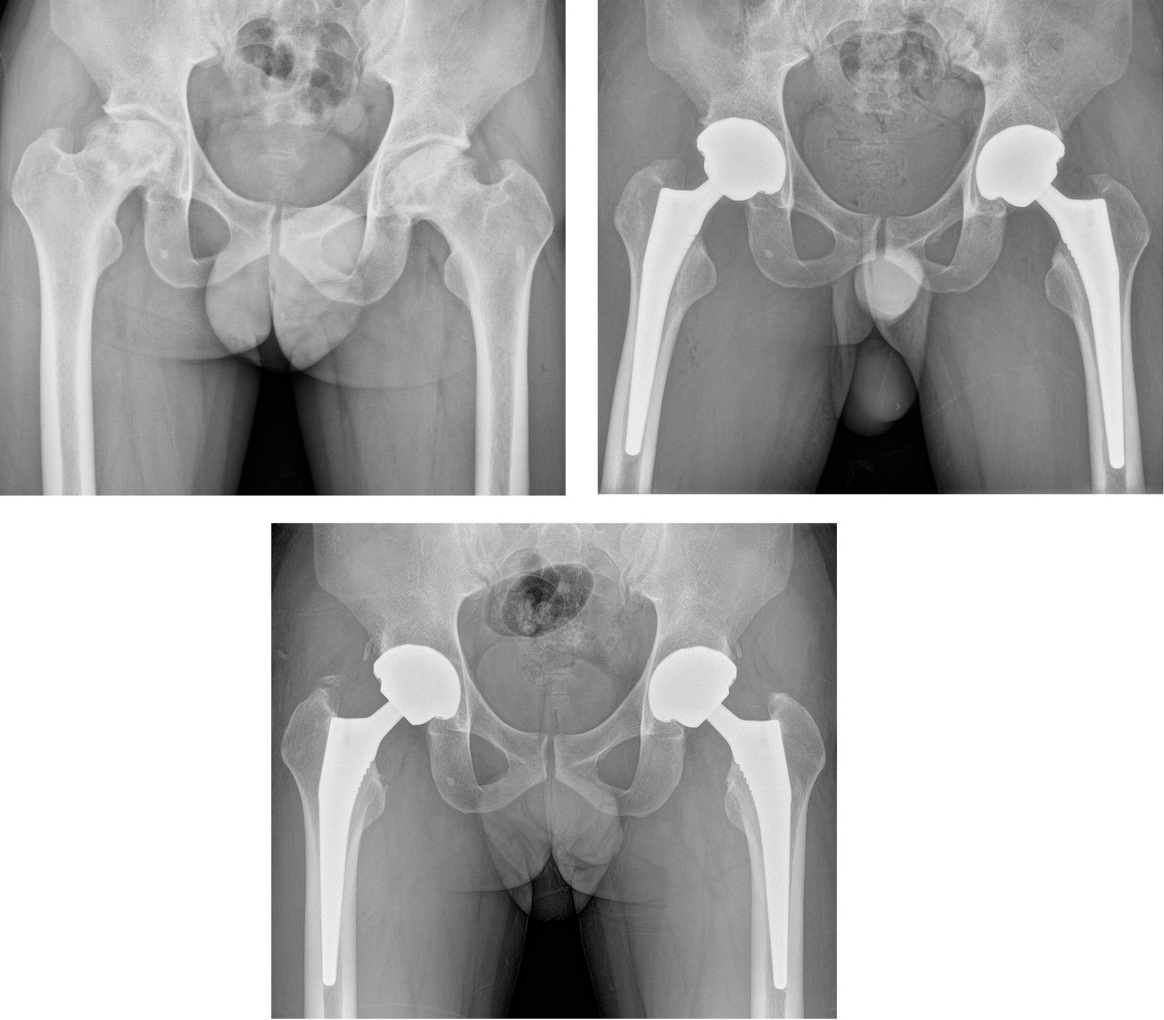
(A) Preoperative hip radiograph of a 43-old man with osteonecrosis of the femoral head involving both hips. (B) Simultaneous bilateral total hip arthroplasty was performed with cementless acetabular components and tapered wedge femoral stems. Closed suction drainage was not used for this case. Calculated blood loss was 1254 mL, and allogenic blood transfusion was not performed. (C) A 4-year postoperative radiograph showed stable implant fixations. The patient was able to participate in daily activities without pain and was satisfied with the surgical outcome.

**Fig 2:**
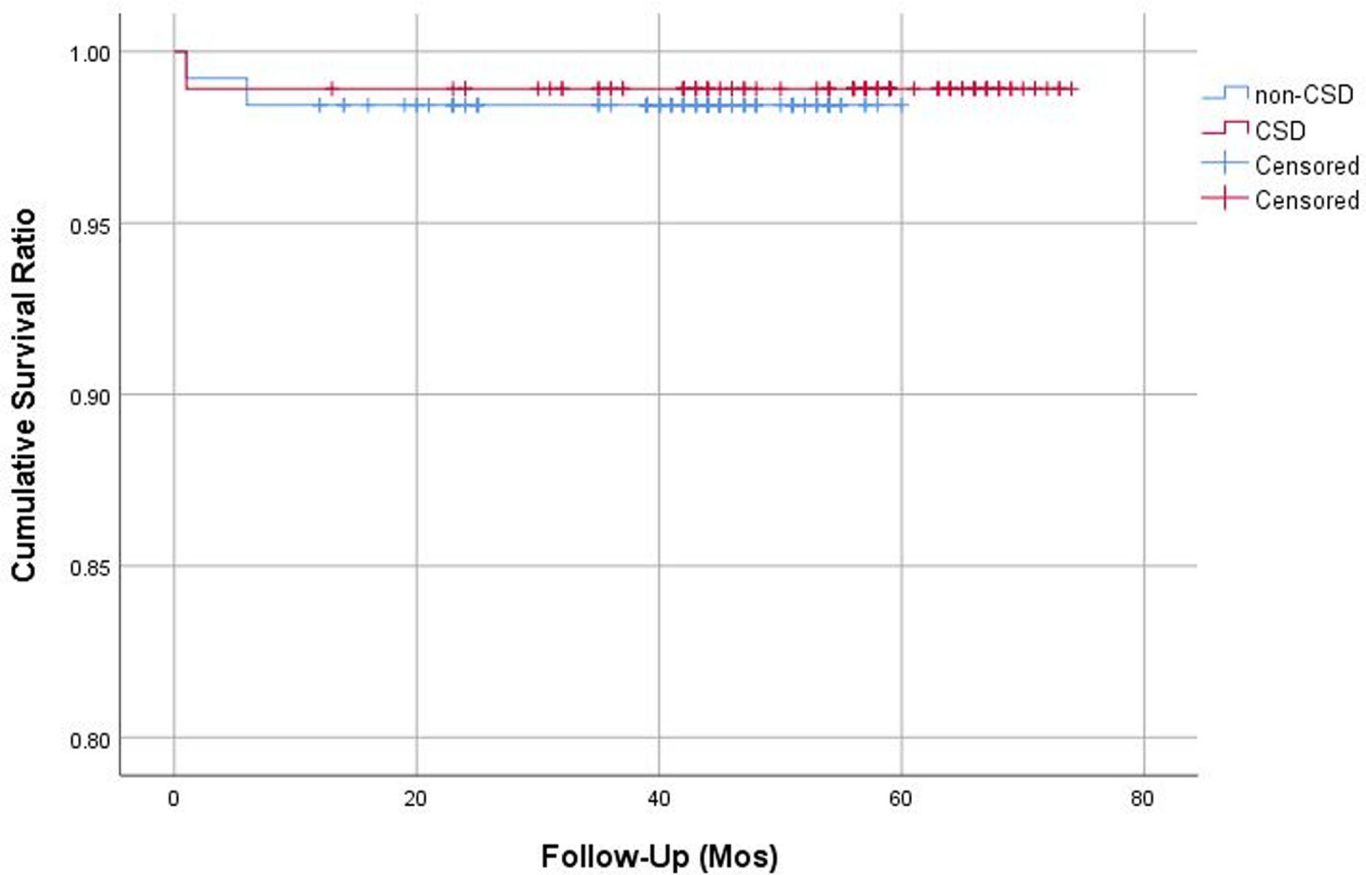
Kaplan-Meier survival curves with ends points of revision for any reason. CSD, closed suction drainage.

No significant difference was found between the CSD and non-CSD groups in HHS, UCLA activity score, patient satisfaction rate, and combined ROM at final follow-up (Table 4) (Fig 1). One periprosthetic joint infection (1.1%) occurred in the CSD group, which was successfully managed by irrigation and debridement and prolonged antibiotic treatment. In the non-CSD group, one arthroscopic iliopsoas tenotomy was performed due to chronic inguinal pain after THA. The femoral component was revised for periprosthetic femoral fracture in one hip (1.1%) in the CSD group and two hips (1.6%) in the non-CSD group. Implant survivorship with an end-point of revision for any reason was 98.9% (95% confidence interval [CI], 96.7–100%) for the CSD group and 98.4% (95% CI, 96.2–100%) for the non-CSD group at 4 years, and the difference was not significant (log-rank, P = 0.766) (Fig 2).

**Table 4:** Final surgical outcomes and complications.

Parameter	CSD	non-CSD	P-value
Harris Hip Score*	92.5 ± 8.3	92.1 ± 8.7	0.775
UCLA activity score†	6 (3–8)	6 (3–8)	0.488
Number of satisfactory hips	86 (93.5%)	122 (95.3%)	0.555
Combined range of motion* (°)	222.3 ± 21.0	219.6 ± 25.3	0.399
Major surgical complication			
Periprosthetic joint infection	1 (1.1%)	0 (0%)	0.418
Dislocation	0 (0%)	0 (0%)	1.000
Periprosthetic femoral fracture	1 (1.1%)	2 (1.6%)	1.000
Aseptic implant loosening	0 (0%)	0 (0%)	1.000
Any secondary hip surgery	2 (2.2%)	3 (2.3%)	1.000
Revision hip arthroplasty			
Acetabular component revision	0 (0%)	0 (0%)	1.000
Femoral component revision	1 (1.1%)	2 (1.6%)	1.000

*The values are given as the mean and the standard deviation.

†The values are given as the median with the range in parentheses. Other values are given as the number of hips with the percentage in parentheses. CSD, closed suction drainage; UCLA, University of California, Los Angeles.

## Discussion

Although SBTHA can be an attractive option for patients with bilateral hip disease, there still remains concerns on the increased blood loss and frequent need for allogenic transfusion. In this study, we reviewed consecutive series of 110 cementless SBTHAs to evaluate the effect of disusing CSD on perioperative blood loss and transfusion. We found that the maximum hemoglobin drop, calculated blood loss, transfusion rate, and the mean units of transfusion were significantly lower in patients who underwent SBTHA without CSD. After disusing CSD, the mean calculated blood loss decreased from 1530 mL to 1190 mL, transfusion rate reduced from 46% to 22%, and the mean units of transfusion decreased from 0.9 to 0.4. Postoperative in-hospital pain scales, wound complication rate, major medical and surgical complication rates, and final clinical scores were not significantly affected by disusing CSD. Revision-free implant survivorships were similar between groups at 4 years (98.9% vs. 98.4%; log-rank, P = 0.766).

There have been controversies whether SBTHA or staged bilateral THA should be performed in patients requiring THA for both hips. Several studies have reported higher incidence of adverse events following SBTHA [16, 31, 32], while others reported similar or better outcomes of SBTHA [6, 7, 10–13]. Berend et al.[16] reported that the incidence of adverse events (71.3% vs. 42.3%) and the mean number of transfusion (0.8 vs. 0.4 units) were significantly higher in the simultaneous group compared to the staged group. However, Romagnoli et al. [3] reported that there was no significant difference in the rates of allogenic transfusion and other postoperative complication between the groups of SBTHA and unilateral THA. The higher incidence of adverse events after SBTHA may be associated with an increased blood loss and transfusion. Not only are hypovolemia and anemia possible life threatening conditions, allogenic blood transfusion is also a known risk factor for postoperative infection, venous thromboembolism, and acute lung injury in TJA [33–36]. Although differences in studies, the transfusion rate after SBTHA is reported to be as high as 50% [15, 17, 33]. Therefore, to perform SBTHA more safely, surgeons should focus on reducing perioperative blood loss and transfusion.

In recent years, there have been great advancements in TJA; improved surgical technique, strict transfusion criteria, standardized thromboembolic prophylaxis, and the use of TXA have reduced the incidence of systemic complications [13, 37]. Of those, TXA is one of the greatest successes in reducing transfusion, and is now considered a standard of care in TJA [38]. Together with other advancements, the widespread use of TXA could have improved SBTHA safety. Recent data supports that SBTHA has a similar risk of systemic complication compared to staged bilateral THA [3, 6, 12, 13]. A meta-analysis by Shao et al. [13] reported that SBTHA was even superior to staged operations in terms of surgical time, thromboembolic event, and major systemic complication. Within our study population, there was no major medical complication observed after SBTHA with administration of intravenous TXA. This result can be partially supported by a recent multicenter study that the use of TXA decreased the risk of blood transfusion in SBTHA [33].

Avoiding the routine use of CSD can be an effective strategy to reduce blood loss in SBTHA. Although CSD has been commonly used in orthopedic surgeries for decades, the necessity of its use in TJA has been increasingly questioned in these days [19]. Theoretically, CSD can block natural tamponade effect in the surgical wound space and cause unnecessary bleeding by applying negative pressure on it. Several clinical studies have reported that CSD causes extra blood loss and elevates transfusion requirement in TJA [20–22, 39–41]. A recent meta-analysis involving 3,186 patients from 20 randomized controlled trials identified that the use of CSD in THA increased the rate of transfusion [21]. Based on these reports, the senior author in this study decided not to use CSD in all primary THAs since June 2015. As a result, the mean blood loss was reduced by an amount of 340 mL and transfusion rate decreased by 52% after disusing CSD in SBTHA. Therefore, the routine use of CSD may not be indicated when performing cementless SBTHA.

Nevertheless, there are controversies on the CSD use in THA. Apart from the systemic effects, CSD was originally intended to reduce local swelling, hematoma, skin tension, and encourage wound healing and rehabilitation [18, 19, 21, 37]. Koyano et al. [18] reported that postoperative local inflammation and pain scores were significantly lower in patients who underwent THA with CSD. In the present study, however, no disadvantage was observed in wound complication rate, early postoperative pain scores, final clinical scores, combined ROM, and satisfaction rate after disusing CSD. These results are supported by other studies reporting no obvious clinical benefits of CSD use in TJA [20–22, 40]. One particular concern regarding SBTHA is the pressure applied to the first surgical wound during the second hip surgery in a lateral position. In the present study, however, there was no difference in wound complications between the first and second hips of SBTHA. No skin necrosis, severe hematoma, or nerve palsy was observed in the first hip in either group. Our findings suggest that even without CSD, there may be no definite adverse effect of compressing the first wound during the second hip procedure in SBTHA.

Hematoma is believed to be associated with bacterial colonization and interfere with the body’s defense mechanism [19], and the use of CSD can be expected to reduce infection. A few studies identified lower infection rates in TJA with CSD [41, 42], while others did not [15, 18, 20–22, 40, 43]. Some authors insist that the use of CSD can lead to postoperative infection as the drain tube can act as a path through which bacteria can reversely penetrate [43]. This can be more problematic when CSD was left in the wound for longer periods. In this study, we could not find a significant difference in overall infection rates between the CSD and non-CSD groups. However, 2 cases of superficial infection occurred only in the CSD group, and prolonged drain hole leakage was found in 2 hips. Convincing evidences regarding the role of CSD on postoperative infection in TJA are still lacking. A prospective study with a large number of cases would be required to identify the effect of disusing CSD on the infection rate after SBTHA.

To our knowledge, this is the first study to report the amount of reduction in postoperative blood loss and allogenic transfusion after disusing CSD in SBTHA. We could calculate the amount of blood loss relatively accurately by using the preoperative and lowest postoperative hemoglobin concentrations. However, there are also several limitations to the present study. First, due to the historical nature of our comparison, there are differences in the time periods of performing SBTHA in two groups. However, the surgical procedures of SBTHA and postoperative managements remained largely unchanged during the 3 years of inclusion period except for the CSD use. Second, since this is a retrospective study, the incidence of wound complications and other adverse events could have been underestimated. Third, majority of the study population included young patients with osteonecrosis of the femoral head. This patient characteristics may have influenced the results of the study. Finally, the number of cases for each group may be insufficient to identify differences in the incidence of rare, but critical complications, such as periprosthetic joint infection and venous thromboembolic events.

## Conclusions

Disusing CSD significantly reduced postoperative blood loss and need for allogenic transfusion without increasing postoperative pain, local wound complications, and major medical and surgical complications in cementless SBTHA with intravenous TXA. This study suggests that the routine use of CSD may not be indicated when performing cementless SBTHA with concurrent administration of intravenous TXA.

## Supporting information

S1 Dataset Data used for analysis. (XLSX)
